# Supplementary material for: Non-Abelian anyon collider
Source: Nat Commun. 2022 Nov 4;13:6660. doi: 10.1038/s41467-022-34329-y (PMC9636162; doi:10.1038/s41467-022-34329-y)
Supplement: Supplementary file 1 — Supplementary Information [file 41467_2022_34329_MOESM1_ESM.pdf]

Supplementary Information: Non-Abelian Anyon Collider

June-Young M. Lee and H.-S. Sim
Department of Physics, Korea Advanced Institute of Science and Technology, Daejeon 34141, Korea

Supplementary Note 1. DERIVATION OF THE NONEQUILIBRIUM CORRELATOR IN EQ. (1)

The non-equilibrium correlator in Eq. (1) is decomposed into a product of correlators of the edges A and B, $\langle \mathcal{T}^\dagger(0)\mathcal{T}(t)\rangle_{\text{neq}} \propto \left\langle [\psi_{\text{A}}^\dagger(0,0)\psi_{\text{A}}(0,t)]_I \right\rangle_{\text{neq}} \left\langle [\psi_{\text{B}}(0,0)\psi_{\text{B}}^\dagger(0,t)]_I \right\rangle_{\text{neq}}$. We here derive the correlator of Edge A.

The tunneling current $I_{\text{A,inj}}$ is generated at QPC_{A} from Edge A_{inj} (the edge directly connected to Source S_{A}) to Edge A by the voltage $V_{\text{A,inj}}$ applied to Source S_{A}. We assume that the weak coupling regime is dominated by tunneling of a single quasiparticle type, described by the tunneling Hamiltonian $H_{\text{A,inj}} = \mathcal{A}(t) + \mathcal{A}^\dagger(t)$,

$$\mathcal{A}(t) = \gamma_{\text{A}} e^{-ie^* V_{\text{A,inj}} t/\hbar} [\psi_{\text{A,inj}}(0,t)\psi_{\text{A}}^\dagger(-d,t)]_I \cdots . \tag{S1}$$

with the tunneling strength γ_{A} and the charge e^* carried by the quasiparticle. At certain filling factors, the quasiparticle is fractionalized into downstream and upstream parts upon its tunneling. We focus on the fractionalized part ψ_{A} moving from QPC_{A} to QPC_{C} along Edge A, since this determines I_{T}, $\langle \delta I_{\text{T}}^2\rangle$, $\langle \delta I_{\text{A}} \delta I_{\text{B}}\rangle$. $\psi_{\text{A,inj}}$ is the corresponding part on Edge A_{inj}. $[\cdots]_{a=I}$ means that $\psi_{\text{A}}^\dagger$ and $\psi_{\text{A,inj}}$ are pair-created from the vacuum fusion channel $a = I$. The other part has negligible contribution to the observables (see Sec. Supplementary Note 4), and omitted as $\cdots$ in Eq. (S1). The anyon ψ_{A} is described by the primary field of the relevant conformal field theory (CFT) with the scaling dimension h_ψ and quantum dimension d_ψ. We will derive the correlator of the anyons on Edge A at the voltage $V_{\text{A,inj}}$,

$$\left\langle [\psi_{\text{A}}(x,t)\psi_{\text{A}}^\dagger(0,0)]_I \right\rangle_{\text{neq}} = e^{\text{Re}[M-1]\frac{I_{\text{A,inj}}}{e^*}|t-\frac{x}{v}|} e^{i\,\text{Im}[M-1]\frac{I_{\text{A,inj}}}{e^*}(t-\frac{x}{v})} \left\langle [\psi_{\text{A}}(x,t)\psi_{\text{A}}^\dagger(0,0)]_I \right\rangle_{\text{eq}} + \text{subleading terms.} \tag{S2}$$

$\left\langle [\psi_{\text{A}}\psi_{\text{A}}^\dagger]_I \right\rangle_{\text{eq}}$ is the equilibrium correlator at $V_{\text{A,inj}} = 0$. v is the anyon velocity. We note that the tunneling current is expressed as $I_{\text{A,inj}} = 2\pi|\gamma_{\text{A}}|^2 e^*(e^* V_{\text{A,inj}}/\hbar)^{4h_\psi - 1}/[d_\psi \Gamma(4h_\psi)]$ when the quasiparticle is fully described by ψ (i.e., not fractionalized). Below we drop the edge index A and focus on $vt - x > 0$; the $vt - x < 0$ case is obtained similarly.

We perform the Keldysh pertubative expansion $\langle [\psi(x,t)\psi^\dagger(0,0)]_I \rangle_{\text{neq}} = \sum_{n=0}^\infty \mathcal{C}_n(x,t)$ over arbitrary orders of the tunneling strength at QPC_{A}. $\mathcal{C}_n(x,t)$ is the $2n$-th order perturbation term proportional to $|\gamma_{\text{A}}|^{2n}$ and $(-i)^{2n}/(2n)!$,

$$\mathcal{C}_n(x,t) = \frac{(-1)^n}{(2n)!}\frac{(2n)!}{n!n!}\int_K dt_1 \cdots dt_{2n} \langle T_K\{[\psi(x,t)\psi^\dagger(0,0)]_I \prod_{i=1}^n \mathcal{A}^\dagger(t_{2i-1})\mathcal{A}(t_{2i})\}\rangle. \tag{S3}$$

We set $\hbar \equiv 1$. $T_K\{\cdots\}$ is the Keldysh time ordering. $\int_K dt_1 \cdots dt_{2n}$ is the time t_i integration on the Keldysh contour. $\prod_{i=1}^n \mathcal{A}^\dagger(t_{2i-1})\mathcal{A}(t_{2i})$ is a combination of n $\mathcal{A}$ and n $\mathcal{A}^\dagger$ chosen from $2n$ H_{inj} operators. The total number of such combinations is $(2n)!/n!n!$. It is obvious that $\mathcal{C}_0(x,t)$ equals the equilibrium correlator $\langle [\psi(x,t)\psi^\dagger(0,0)]_I\rangle_{\text{eq}}$.

We compute $\mathcal{C}_n$ in the regime of large $e^* V_{\text{inj}}$. The integral in $\mathcal{C}_n$ is dominated by the time windows of t_i's where anyons from n $\mathcal{A}^\dagger(t_{2i-1})$'s and those from n $\mathcal{A}(t_{2i})$'s pairwise overlap on Edge A, i.e., the anyons in each pair are located within short distance $\hbar v/(e^* V_{\text{inj}})$; when $e^* V_{\text{inj}}$ is the largest energy, it is a good approximation to set $t_{2i-1} \simeq t_{2i'}$ (pairing time indices $2i-1$ and $2i'$). We set $t_{2i-1} \simeq t_{2i}$ and order them as $t_1 \simeq t_2 < t_3 \simeq t_4 < \cdots$, without loss of the generality, with multiplying the number $n!$ of the equivalent ways of the pairings to the integral. The Keldysh index at time t_j is $\eta_j = +1$ for the forward branch of the Keldysh contour and $\eta_j = -1$ for the backward branch. Then

$$\mathcal{C}_n(x,t) \simeq |\gamma_{\text{A}}|^{2n}\frac{(-1)^n}{n!}\sum_{\eta_j=\pm}\prod_j \eta_j \int_{t_{2i-1}\simeq t_{2i}} dt_1\cdots dt_{2n} e^{i\omega\sum_i (t_{2i-1}-t_{2i})}\mathcal{F}^{\vec{\eta}}, \tag{S4}$$

where $\omega = e^* V_{\text{inj}}/\hbar$. $\mathcal{F}^{\vec{\eta}}$ is the conformal block of the primary fields ψ's,

$$\mathcal{F}^{\vec{\eta}} \equiv \frac{e^{-i\omega\sum_i(t_{2i-1}-t_{2i})}}{|\gamma_{\text{A}}|^{2n}} \langle T_K\{[\psi(x,t)\psi^\dagger(0,0)]_I \prod_{i=1}^n \mathcal{A}^\dagger(t_{2i-1}^{\eta_{2i-1}})\mathcal{A}(t_{2i}^{\eta_{2i}})\}\rangle. \tag{S5}$$

Here the factor $e^{-i\omega\sum_i(t_{2i-1}-t_{2i})}/|\gamma_\mathrm{A}|^{2n}$ cancels the corresponding factors in $\mathcal{A}$'s so that $\mathcal{F}^{\vec\eta}$ contains only the anyon fields. In the conformal block $\mathcal{F}^{\vec\eta}$, $\psi(x,t)$ and $\psi^\dagger(0,0)$ are the main fields of the correlator, while the others $\psi(-d,t_{2i-1}^{\eta_{2i-1}})$'s and $\psi^\dagger(-d,t_{2i}^{\eta_{2i}})$'s in $\mathcal{A}^\dagger$'s and $\mathcal{A}$'s describe anyons injected to Edge A through $\mathrm{QPC_A}$ by $V_{\mathrm{A,inj}}$.

For the injected anyons to be correlated with the anyons described by the main fields $\psi(x,t)$ and $\psi^\dagger(0,0)$, they should be injected before the time 0 or t. Then, after the Keldysh ordering, the conformal block has the ordering of

$$\mathcal{F}^{\vec\eta} \propto \langle(\mathcal{A},\,\mathcal{A}^\dagger\text{s with } \eta_j=-1)[\psi(x,t)\psi^\dagger(0,0)]_I(\mathcal{A},\,\mathcal{A}^\dagger\text{s with } \eta_j=1)\rangle.$$

In the case where $\eta_j=-1$ for all j's, for example, $\mathcal{F}^{\vec\eta}$ has the following form and diagramatic representation,

$$\mathcal{F}^{(-1,\cdots,-1)} = \langle[\psi(-d,t_1)\psi^\dagger_{\mathrm{inj}}(0,t_1)]_I[\psi_{\mathrm{inj}}(0,t_2)\psi^\dagger(-d,t_2)]_I[\psi(-d,t_3)\psi^\dagger_{\mathrm{inj}}(0,t_3)]_I\cdots[\psi(x,t)\psi^\dagger(0,0)]_I\rangle$$

$$= d_\psi^{-(n+\frac{1}{2})}$$ 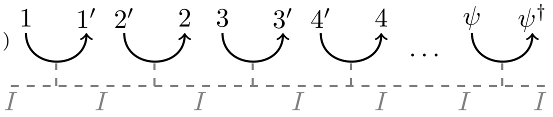

(S6)

where the head (resp. tail) of the arrow with numbers j, j' depicts the creation (resp. annihilation) operator of the square braket at t_j. The numbers without (with) the prime symbol depict anyons on Edge A ($\mathrm{A_{inj}}$). The symbol I means the vacuum fusion channel. We use the normalization convention of the diagrams in Ref. [S1].

For further computation, we express the conformal block $\mathcal{F}^{\vec\eta}$ as a linear combination [S2] of the fusion bases $\mathcal{G}^{\vec\eta}_{\vec a,\vec b}$'s in which the fields at similar spacetime $t_{2i-1}\simeq t_{2i}$ on the same edge fuse first to an intermediate fusion state a_i,

$$\mathcal{G}^{\vec\eta}_{\vec a,\vec b} = \Big\langle[[[[[\psi(-d,t_1)\psi^\dagger(-d,t_2)]_{a_1=b_1}[\psi^\dagger_{\mathrm{inj}}(0,t_1)\psi_{\mathrm{inj}}(0,t_2)]_{a_2}]_{b_2}[\psi(-d,t_3)\psi^\dagger(-d,t_4)]_{a_3}]_{b_3}\cdots]_{b_{2n}}[\psi(x,t)\psi^\dagger(0,0)]_{a_{2n+1}}]_I\Big\rangle$$

$$= d_\psi^{-(n+\frac{1}{2})}$$ 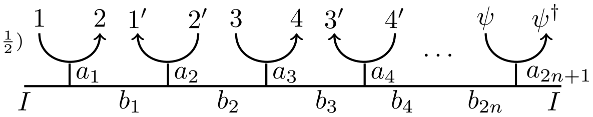

. (S7)

$[[\cdots]_{b_j}[\cdots]_{a_{j+1}}]_{b_{j+1}}$ means the fusion of intermediate states b_j and a_{j+1} into b_{j+1}. The operator product expansion [S3] (OPE) is applied at $t_{2i-1}\simeq t_{2i}$, to obtain $\mathcal{G}^{\vec\eta}_{\vec a,\vec b}\propto(t_{2i-1}-t_{2i})^{h_{a_{2i-1}}+h_{a_{2i}}-4h_\psi}$. h_a is the scaling dimension of the primary field a. At large V_{inj}, $\mathcal{F}^{\vec\eta}$ is determined by the basis $\mathcal{G}^{\vec\eta}_{\vec I,\vec I}$ with $a_j=b_j=I$ where all the intermediate states are the vacuum field of the lowest scaling dimension $h_I=0$. One has $\mathcal{G}^{\vec\eta}_{\vec I,\vec I}\simeq\langle[\psi_\mathrm{A}(x,t)\psi^\dagger_\mathrm{A}(0,0)]_I\rangle_{\mathrm{eq}}\prod_i[\epsilon+i\chi_{\eta_{2i-1},\eta_{2i}}(t_{2i-1}-t_{2i})]^{-4h_\psi}$ with $\chi_{\eta_{2i-1},\eta_{2i}}=[(\eta_{2i-1}+\eta_{2i})\mathrm{sgn}(t_{2i-1}-t_{2i})-(\eta_{2i-1}-\eta_{2i})]/2$. ϵ is a positive infinitesimal cutoff.

To compute the overlap of $\mathcal{F}^{\vec\eta}$ and $\mathcal{G}^{\vec\eta}_{\vec I,\vec I}$, we glue the diagrams $\mathcal{F}^{\vec\eta}$ and $\mathcal{G}^{\vec\eta}_{\vec I,\vec I}$, reversing arrow directions in the diagram $\mathcal{G}^{\vec\eta}_{\vec I,\vec I}$ and connecting the arrow of index j of $\mathcal{G}^{\vec\eta}_{\vec I,\vec I}$ with the arrow of the same index of $\mathcal{F}^{\vec\eta}$. When different connections j_1 and j_2 cross, connection j_1 is drawn on top of j_2 if $x_{j_1}-vt_{j_1}>x_{j_2}-vt_{j_2}$ [S4]. The overlap depends on the Keldysh indices η_i. In the case of $\eta_j=-1$ for all j's, the gluing of Eqs. (S6) and (S7) has $n+1$ unlinked loops,

$$\frac{1}{d_\psi^{2n+1}}$$ 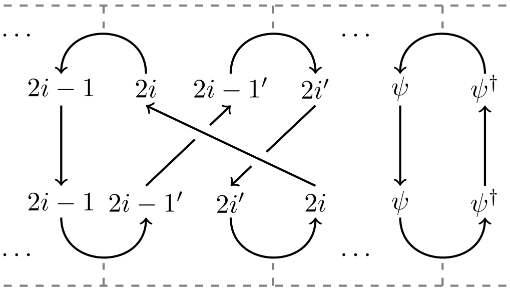

$$= \frac{1}{d_\psi^n}.$$ (S8)

Each loop is composed of either connections $(2i-1)'$, $(2i)'$, $(2i-1)$ and $(2i)$ describing an (particle-like) anyon on Edge A injected at $\mathrm{QPC_A}$ and the remaining (hole-like) anyon on Edge $\mathrm{A_{inj}}$ at time $t_{2i-1}\simeq t_{2i}$, or connections $\psi(x,t)$ and $\psi^\dagger(0,0)$ describing the main fields of the correlator. All the loops are unlinked, and each loop equally contributes to the overlap. Hence the conformal block $\mathcal{F}^{\vec\eta}$ does not involve anyon braiding in the case of $\eta_j=-1$ for all j's.

We next examine the case where $\eta_{2i-1}=\eta_{2i}=1$ for some i. Then in $\mathcal{F}^{\vec\eta}$, after the Keldysh ordering, $\mathcal{A}^\dagger(t^+_{2i-1})$ and $\mathcal{A}(t^+_{2i})$ are placed right of the anyon fields having $\eta_j=-1$, the main fields $\psi(x,t)$ and $\psi^\dagger(0,0)$, and the fields having

$\eta_j = 1$ at $t_j > t_{2i-1} \simeq t_{2i}$. No anyon field is placed between $\mathcal{A}^\dagger(t^+_{2i-1})$ and $\mathcal{A}(t^+_{2i})$ since $t_{2i-1} \simeq t_{2i}$. In the gluing of $\mathcal{F}^{\vec{\eta}}$ and $\mathcal{G}^{\vec{\eta}}$, the loop composed of connections $\mathcal{A}^\dagger(t^+_{2i-1})$ and $\mathcal{A}(t^+_{2i})$ is trivially unlinked from all the other loops, because $\mathcal{A}^\dagger(t^+_{2i-1})$ and $\mathcal{A}(t^+_{2i})$ always together cross some other connections so that the crossings are trivially loosened. Similarly, any loop is trivially unlinked from all the others. No braiding happens among the injected anyons.

When $\eta_{2i-1} = -\eta_{2i}$ for some i and $0 < vt_{2i-1} + d \simeq vt_{2i} + d < vt - x$, nontrivial links by braiding happen in the overlap between $\mathcal{F}^{\vec{\eta}}$ and $\mathcal{G}^{\vec{\eta}}_{\vec{I},\vec{I}'}$. This is shown for $\eta_{2i} = -\eta_{2i-1} = 1$, for which the overlap is graphically represented,

$$\frac{1}{d_\psi^{2n+1}} \qquad \text{(S9)}$$

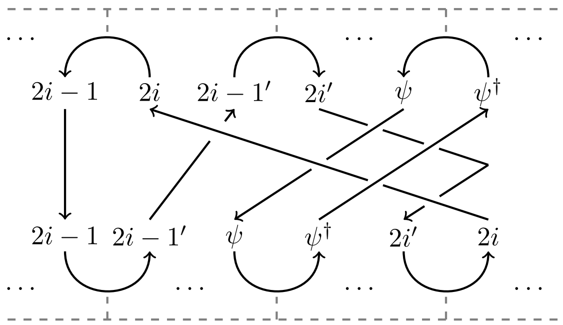

Here $[\psi(x,t)\psi^\dagger(0,0)]_I$ is sandwiched by $\mathcal{A}^\dagger(t^\pm_{2i-1})$ and $\mathcal{A}(t^\mp_{2i})$ in the Keldysh ordering of $\mathcal{F}^{\vec{\eta}}$. In computing the overlap, $\mathcal{A}(t_{2i})$ has to move to the left of ψ and $\psi^\dagger$ as in the operator ordering of $\mathcal{G}^{\vec{\eta}}_{\vec{I},\vec{I}'}$. This is done with crossing of connections in Eq. (S9). When $0 < vt_{2i-1} + d \simeq vt_{2i} + d < vt - x$, the crossings are drawn as follows. Connection $\psi^\dagger$ is on top of all the other connections, connections $(2i-1)$ and $(2i)$ are on top of all the other connections except $\psi^\dagger$, connection ψ is on top of $(2i-1)'$ and $(2i)'$ and below $\psi^\dagger$, $(2i-1)$ and $(2i)$; connections $(2i-1)'$, $(2i)'$, ψ, $(2i-1)$, $(2i)$, and $\psi^\dagger$ are placed in order from bottom to top. Hence the two loops, one composed of the connections ψ and $\psi^\dagger$ for the main anyon fields of the correlator, and the other of the connections $(2i-1)'$, $(2i)'$, $(2i-1)$, $(2i)$ for an anyon generated by the tunneling at QPC_A, are linked in a non-contractible way. Untying the linked loops in Eq. (S9) into the two unlinked ones in Eq. (S8) indicates that the overlap is proportional to the monodromy M of the anyons. All the other cases of $\eta_{2i-1} = -\eta_{2i}$ for some i, the overlap is evaluated similarily. Collecting the results for all the Keldysh indices,

$$\mathcal{F}^{\vec{\eta}} \simeq \frac{1}{d_\psi^n} \prod_i a_{\eta_{2i-1},\eta_{2i}}(vt_{2i}+d)\mathcal{G}^{\vec{\eta}}_{\vec{I},\vec{I}'}, \qquad a_{\eta,\eta'}(v\bar{t}) = \begin{cases} M, & \text{if } 0 < v\bar{t} < vt - x \ \& \ \eta = -\eta' = -1 \\ M^*, & \text{if } 0 < v\bar{t} < vt - x \ \& \ \eta = -\eta' = 1 \\ 1 & \text{otherwise.} \end{cases} \qquad \text{(S10)}$$

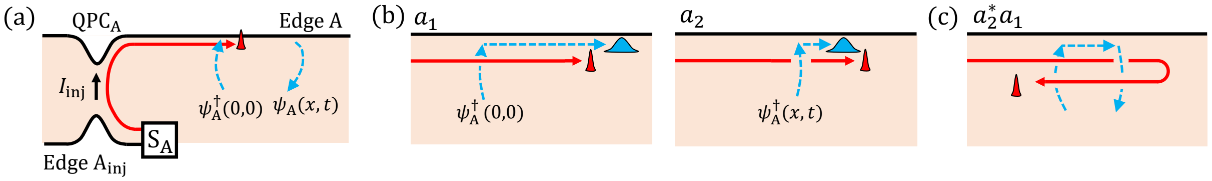

Supplementary Fig. 1. A process contributing to $\mathcal{C}_1(x,t)$. (a) An anyon is injected from Edge A_inj to Edge A by tunneling at QPC_A. This is described by $\mathcal{A}^\dagger(t_{2i-1})$ and $\mathcal{A}(t_{2i})$ in Eq. (S9). As biased by the large voltage V_inj, the anyon has spatial uncertainty $\hbar v/(e^* V_\text{inj})$, hence, $t_{2i-1} \simeq t_{2i}$; its spatial uncertainty and propagation are depicted by red peaks and red solid arrows. Creation and annihilation of another anyon by the main fields $\psi^\dagger_\text{A}(0,0)$ and $\psi_\text{A}(x,t)$ of the correlator are shown by blue dashed arrows. We consider $0 < vt_{2i-1} + d \simeq vt_{2i} + d < vt - x$ and $x, 0 > -d$; $x = -d$ is the location of QPC_A on Edge A. (b) This process is the interference $a_2^* a_1$ of two subprocesses a_1 and a_2. In a_1 the blue anyon [described by $\psi^\dagger_\text{A}(0,0)$] is created at position 0 and time 0 before the red anyon [by $\mathcal{A}^\dagger(t_{2i-1})$] passes the position. In a_2, the blue anyon [by $\psi^\dagger_\text{A}(x,t)$] is created at x and t after the red anyon [by $\mathcal{A}^\dagger(t_{2i})$] passes x. (c) The interference $a_2^* a_1$ is described by merging a_1 and the time reversal of a_2. It shows the red loop and the blue loop linked by braiding. This corresponds to the two linked loops of the overlap diagram in Eq. (S9).

Plugging this into Eq. (S4) and taking summation over all the η_i's and integration over $\frac{t_{2i-1}+t_{2i}}{2}$'s, we get

$$\begin{aligned}\mathcal{C}_n(x,t) \simeq & |\gamma_{\rm A}|^{2n}\frac{(M-1)^n}{d_\psi^n n!}(t-\frac{x}{v})^n\langle[\psi(x,t)\psi^\dagger(0,0)]_I\rangle_{\rm eq}\prod_i\int d(t_{2i-1}-t_{2i})\frac{e^{i\omega(t_{2i-1}-t_{2i})}}{[\epsilon+i(t_{2i-1}-t_{2i})]^{4h_\psi}} \\ = & \frac{1}{n!}[(M-1)(t-\frac{x}{v})\frac{I_{\rm inj}}{e^*}]^n\langle[\psi(x,t)\psi^\dagger(0,0)]_I\rangle_{\rm eq}.\end{aligned}$$

Doing the resummation $\left\langle[\psi(x,t)\psi^\dagger(0,0)]_I\right\rangle_{\rm neq} = \sum_{n=0}^\infty \mathcal{C}_n(x,t)$, we obtain Eq. (S2). A physical process contributing to $\mathcal{C}_1(x,t)$ is shown in Supplementary Fig. 1 as an example. Equation (S2) is valid at long time $|t-x/v| \gg \hbar/e^* V_{\rm inj}$. And the perturbative expansion $\sum_{n=0}^\infty \mathcal{C}_n(x,t)$ converges in the weak-backscattering regime of the QPC.

We emphasize that in Eq. (S2), the monodromy M appears together with the trivial term 1. The combined factor $M-1$ originates from the fact that in every perturbation order, partial cancellation of the terms happens between different Keldysh indices in the domain of $0 < vt_{2i-1}+d \simeq vt_{2i}+d < vt-x$. The combined factor or the partial cancellation is understood, considering the collider of free electrons in the integer quantum Hall regime. For free electrons, braiding is a trivial process, hence, Eq. (S9) is described by a disconnected Feynmann diagram (a vacuum bubble diagram) having $M=1$. This disconnected diagram does not contribute to observables for free electrons, since a partner disconnected diagram (a partner vacuum bubble) having the trivial factor 1 pairwise appears and fully cancels the diagram, $M-1=0$, according to the linked cluster theorem of the many-body perturbation theory. By contrast, for anyons, the two diagrams, one giving the nontrivial monodromy $M \neq 1$ and its partner giving the trivial factor 1, pairwise appear and cancel each other only partially, resulting in the combined factor $M-1 \neq 0$. Anyon processes involving the partial cancellation are called topological vacuum bubbles in Refs. [S5, S6].

Supplementary Note 2. SUBLEADING CONTRIBUTIONS TO THE CORRELATOR AND CURRENTS

We discuss and estimate certain subleading terms in Eqs. (1) and (2) [also in Eq. (S2)], a subleading term corresponding to the coventional collision process of free electrons, and another subleading term that regularizes the diverging behavior of P_- in the cases of the non-Abelian anyons having ${\rm Im}[M]=0$.

A. Direct collision

We consider the direct collision process, in which the particles injected at $\text{QPC}_{\rm A/B}$ by the voltage $V_{\rm A/B,inj}$ directly tunnel at $\text{QPC}_{\rm C}$. For estimation of its contribution to the nonequilibrium correlator in Eq. (1), it is sufficient to consider the lowest-order perturbation in the tunneling strength at $\text{QPC}_{\rm A/B}$.

This process has the configuration of time and Keldysh indices of $t_1+d/v \simeq 0$, $t_2+d/v \simeq t-x/v$, and $\eta_1 = -\eta_2 = -1$. In the fusion basis $\mathcal{G}^{\vec\eta}_{\vec I,\vec I}$ for this configuration, $\psi(-d,t_1)$ and $\psi^\dagger(0,0)$ fuse to the vacuum channel, and $\psi(x,t)$ and $\psi^\dagger(-d,t_2)$ also fuse to the vacuum channel. The overlap of $\mathcal{F}^{\vec\eta}$ and $\mathcal{G}^{\vec\eta}_{\vec I,\vec I}$ for the lowest-order perturbation is shown as

$\frac{1}{d_\psi^3}$ 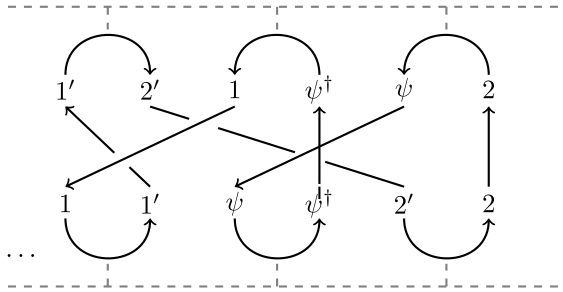 $= \frac{e^{-i2\pi h_\psi}}{d_\psi^2},$ (S11)

where $e^{-i2\pi h_\psi}$ is the topological spin of the anyon. The process contributes to the nonequilibrium correlator as

$$\frac{d_\psi^{-2}|\gamma_{\rm A}|^2 e^{-i2\pi h_\psi}}{[\epsilon-i(t-x/v)]^{2h_\psi}}\int dt_1\frac{e^{i\omega t_1}}{[\epsilon+i(t_1+d/v)]^{2h_\psi}}\int dt_2\frac{e^{-i\omega t_2}}{[\epsilon-i(t_2-t+(d+x)/v)]^{2h_\psi}} = \frac{4\pi^2|\gamma_{\rm A}|^2\omega^{4h_\psi-2}}{\Gamma(2h_\psi)^2 d_\psi^2}\frac{e^{-i\omega(t-x/v)}}{[\epsilon+i(t-x/v)]^{2h_\psi}}. \tag{S12}$$

We notice that the contribution smoothly recovers the free electron result of integer quantum Hall edges as $h_\psi \to 1/2$ and $d_\psi \to 1$. This shows that this process corresponds to the conventional collision process of free electrons.

We further estimate the contribution of this process to $I_{\rm T}$, $\langle \delta I_{\rm T}^2\rangle$, and $\langle \delta I_{\rm A}\delta I_{\rm B}\rangle$ as $O\Big(V_{\rm A/B,inj}^{4\delta-3}\Big)$. We compare it with the leading contribution from the time-domain interference involving braiding,

$$\frac{\text{Contribution from the direct collision process}}{\text{Contribution from the leading term in Eq. (2)}} = O\Big(\big(\frac{\hbar}{e^{*2}}\frac{I_{\rm inj}}{V_{\rm inj}}\big)^{2-2\delta}\Big). \tag{S13}$$

Hence, for $\delta < 1$, the collision process is sub-dominant in comparison with the time-domain interference, as discussed in the main text. We remark that because of the exponential factor $e^{-i\omega(t-x/v)}$ in Eq. (S12), the contribution of the direct collision process to $I_{\rm T} = e^* \int_{-\infty}^{\infty} dt \left\langle \left[\mathcal{T}^\dagger(0), \mathcal{T}(t)\right]\right\rangle_{\rm neq}$ and $\langle \delta I_{\rm T}^2\rangle = e^{*2}\int_{-\infty}^{\infty} dt \left\langle \left\{\mathcal{T}^\dagger(0), \mathcal{T}(t)\right\}\right\rangle_{\rm neq}$ is mainly determined by the short time window of $t \simeq 0$. Braiding cannot happen in the direct process, since it requires other time windows of $0 < t_{2i-1} + d/v \simeq t_{2i} + d/v < t$ or $t < t_{2i-1} + d/v \simeq t_{2i} + d/v < 0$.

B. Subleading braiding process

We consider non-Abelian anyons having ${\rm Im}[M] = 0$. In this particular case, the time-domain interference involving the braiding in Eq. (S9) provides the dominant contribution to Eq. (1), but its contribution to $I_{\rm T}$ in Eq. (2) vanishes, leading to the divergence of P_- in the main text. The divergence is regularized by the process that gives a subleading contribution to Eq. (1) and the biggest contribution to $I_{\rm T}$ when ${\rm Im}[M] = 0$. We below discuss this subleading process.

There are other time-domain interference processes identical to the braiding process of Eq. (S9), having the pairings of $t_{2i'-1} \simeq t_{2i'}$ in the time configuration, except that its fusion basis $\mathcal{G}^{\vec\eta}_{\vec a,\vec b}$ differs from the basis $\mathcal{G}^{\vec\eta}_{\vec I,\vec I}$ of Eq. (S9). Among those processes, we focus on the one in which the main fields ψ and $\psi^\dagger$ of the correlator is fused into a fusion state $a_{2n+1} = a \neq I$ different from the vacuum I while the fusion state a_{2i-1} of an injected anyon, described by $\mathcal{A}^\dagger(t_{2i-1})$ and $\mathcal{A}(t_{2i})$ at $t_{2i-1} \simeq t_{2i}$, is in the fusion channel $a_{2i-1} = \bar a$ on the anti-anyon of a; all the other fusion states $a_{j\neq 2i-1,2n+1}$ are the vacuum I, and hence, the intermediate fusion states b_i are either a ($b_{2i-1} = \cdots = b_{2n} = a$) or the vacuum ($b_1 = b_2 = \cdots = b_{2i-2} = I$). The overlap of this fusion basis $\mathcal{G}^{\vec\eta}_{\vec a,\vec b}$ and the conformal block $\mathcal{F}^{\vec\eta}$ is depicted,

$\dfrac{1}{d_\psi^{2n+1}}$ 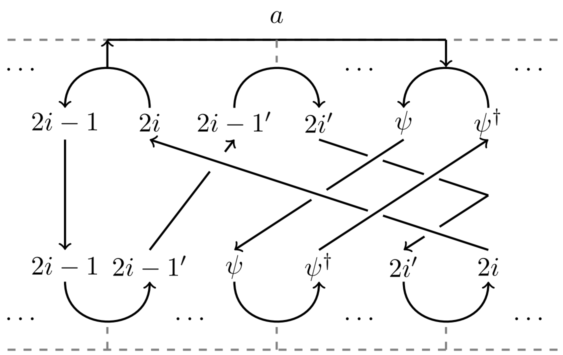 (S14)

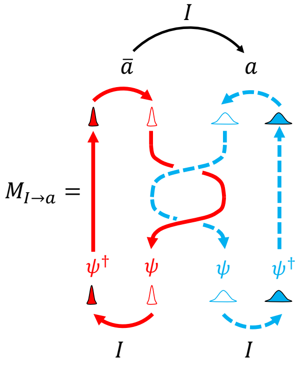

Supplementary Fig. 2. Monodromy $M_{I\to a}$. Two particle-hole pairs of ψ anyons are initially split from the vacuum. After the braiding, they finally fuse into channels $\bar a$ and a. The transition amplitude is $M_{I\to a}$. The monodromy M discussed in the main text is $M_{I\to I}$.

showing that the two loops, one composed of the connections ψ and $\psi^\dagger$ for the main anyon fields of the correlator, and the other of the connections $(2i-1)'$, $(2i)'$, $(2i-1)$, $(2i)$ for an anyon injected through $\mathrm{QPC_A}$, are linked in a non-contractible way, similarly to Eq. (S9). However, untying the linked loops in Eq. (S14) into two unlinked ones indicates that the overlap is proportional to another monodromy $M_{I\to a}$ of the anyons, which differs from the monodromy M involved in Eq. (S9) and discussed in the main text. $M_{I\to a}$ is depicted in Supplementary Fig. 2.

The value of $M_{I\to a}$ depends on non-Abelian anyons. For $\mathrm{SU}(2)_k$ anyons of the anti-Read-Rezayi (ARR) state at the level k, the fusion rule for $j=1/2$ anyons is $\frac{1}{2}\times\frac{1}{2}=0+1$, hence, there are two possible a's, $j=0$ and $j=1$. The fusion channel $j=0$ is the vacuum field I, and the case of $M_{I\to a=I}$ is discussed in Eq. (S9). The case of the $j=1$ fusion channel has the monodromy $M_{I\to j=1}=\frac{(e^{-2\pi i/(2+k)}-1)[\Gamma(k/(2+k))]^2}{\Gamma((-1+k)/(2+k))\Gamma((1+k)/(2+k))}$. The anti-Paffian state at $\nu=5/2$ (the ARR state of level $k=2$) has $M_{I\to j=1}=-e^{i\pi/4}$ and the ARR state of level $k=3$ at $\nu=12/5$ has $M_{I\to j=1}=\frac{(e^{-2\pi i/5}-1)[\Gamma(3/5)]^2}{\Gamma(2/5)\Gamma(4/5)}$. On the other hand, for the Ising anyon in the particle-hole symmetric Pfaffian state, the fusion rule is $\sigma\times\sigma=I+\Psi$, hence, there are also two possible a's, the vacuum field I and the fermion channel Ψ. The case of the fermion channel Ψ has the monodromy $M_{I\to\Psi}=e^{i\pi/4}$. For all these non-Abelian anyons, $\mathrm{Im}[M_{I\to a}]\neq 0$, so the time-domain interference process involving the braiding in Supplementary Fig. 2 provides non-vanishing contribution to I_T.

We compute the contribution from the process in Eq. (S14) to the n-th Keldysh perturbation expansion term $\mathcal{C}_n(x,t)$ of the non-equilibrium correlator $\left\langle[\psi_\mathrm{A}(x,t)\psi_\mathrm{A}^\dagger(0,0)]_I\right\rangle_\mathrm{neq}$. The contribution is expressed as

$$\begin{aligned}&\frac{(M-1)^{n-1}}{(n-1)!}[\frac{I_\mathrm{inj}}{e^*}(t-\frac{x}{v})]^{n-1}|\gamma_\mathrm{A}|^2\frac{M_{I\to a}}{d_\psi}\int d(t_{2i-1}-t_{2i})\frac{e^{i\omega(t_{2i-1}-t_{2i})}}{[\epsilon+i(t_{2i-1}-t_{2i})]^{4h_\psi-h_a}}\\&\times\frac{1}{[\epsilon+i(t-x/v)]^{2h_\psi-h_a}}\int_{-d/v}^{t-(x+d)/v}d\tilde{t}\frac{1}{[\epsilon+i(\tilde{t}-t+(x+d)/v)]^{h_a}}\frac{1}{[\epsilon+i(\tilde{t}+d/v)]^{h_a}},\end{aligned}\tag{S15}$$

where $\tilde{t}=(t_{2i-1}+t_{2i})/2$. In the derivation of this expression, we have multiplied the factor n, since there are n possibilities of choosing a_{2i-1} among a_1, a_3, $\cdots$, a_{2n-1}, integrated out all the time indices t_j's except the times t_{2i-1} and t_{2i} of the anyons fused into the channel a, applied OPE at $t_{2i-1}\simeq t_{2i}$ for connections $(2i-1)$ and $(2i)$, and then used the three-point function of the primary fields [S3]. Performing the integration over $t_{2i-1}-t_{2i}$ and $\tilde{t}$, we obtain

$$\frac{(M-1)^{n-1}}{(n-1)!}[\frac{I_\mathrm{inj}}{e^*}(t-\frac{x}{v})]^{n-1}\frac{M_{I\to a}}{d_\psi}\frac{2\pi|\gamma_A|^2\omega^{4h_\psi-h_a-1}}{\Gamma(4h_\psi-h_a)}\frac{\Gamma(1-h_a)^2}{\Gamma(2-2h_a)}(t-\frac{x}{v})^{1-2h_a}\frac{1}{[\epsilon+i(t-x/v)]^{2h_\psi-h_a}}.\tag{S16}$$

Collecting this contribution to $\mathcal{C}_n(x,t)$ for different n's and doing the resummation of the contributions over n, we obtain the contribution from the process in Eq. (S14) and Supplementary Fig. 2 to the nonequilibrium correlator in Eq. (S2),

$$M_{I\to a}e^{(M-1)\frac{I_\mathrm{inj}}{e^*}(t-x/v)}\frac{\Gamma(1-h_a)^2\Gamma(4h_\psi)}{\Gamma(2-2h_a)\Gamma(4h_\psi-h_a)}\frac{I_\mathrm{inj}}{e^*}(e^*V_\mathrm{inj}/\hbar)^{-h_a}\frac{(t-x/v)^{1-2h_a}}{[\epsilon+i(t-x/v)]^{2h_\psi-h_a}}\tag{S17}$$

at $t-x>0$. This subleading contribution results in a term of $O\Big(I_\mathrm{A/B,inj}^{2\delta+h_a-1}V_\mathrm{A/B,inj}^{-h_a}\Big)$ in I_T and $\langle\delta I_\mathrm{T}^2\rangle$. It is compared with the contribution from the leading term of Eq. (S2) originating from the process in Eq. (S9),

$$\frac{\text{Contribution from the process of }\mathcal{G}^{\vec{\eta}}_{\vec{a},\vec{b}}|_{a_{2n+1}=a,a_{2i-1}=\bar{a}}\text{ to }\langle\delta I_\mathrm{T}^2\rangle}{\text{Contribution from the leading term of Eq. (2) to }\langle\delta I_\mathrm{T}^2\rangle}=O\Big((\frac{\hbar}{e^{*2}}\frac{I_\mathrm{A/B,inj}}{V_\mathrm{A/B,inj}})^{h_a}\Big).\tag{S18}$$

As $h_a>0$, the process involving the monodromy $M_{I\to a}$ indeed gives smaller contribution to $\langle\delta I_\mathrm{T}^2\rangle$ than the leading term of Eq. (S2). The value of h_a is $h_{j=1}=2/(k+2)$ for $\mathrm{SU}(2)_k$ anyons and $h_\Psi=1/2$ for Ising anyons.

Similarly, for anyons having $\mathrm{Im}[M]\neq 0$, the process involving $M_{I\to a}$ negligibly contributes to I_T in comparison with the leading term of Eq. (S2), as the ratio of their contributions is $O\Big((\frac{\hbar}{e^{*2}}\frac{I_\mathrm{A/B,inj}}{V_\mathrm{A/B,inj}})^{h_a}\Big)$ as in Eq. (S18). For non-Abelian anyons having $\mathrm{Im}[M]=0$, however, this process gives the leading contribution to I_T (bigger than the contribution from the direct collision in Eq. (S12)), therefore, $e^*I_\mathrm{T}/\langle\delta I_\mathrm{T}^2\rangle\sim O\Big((\frac{\hbar}{e^{*2}}\frac{I_\mathrm{A/B,inj}}{V_\mathrm{A/B,inj}})^{h_a}\Big)$, resulting in the divergence of P_-.

Supplementary Note 3. DERIVATION OF CROSS CORRELATION $\langle \delta I_{\mathbf{A}} \delta I_{\mathbf{B}} \rangle$

We derive the cross correlation $\langle \delta I_{\rm A} \delta I_{\rm B} \rangle$ at zero temperature. Charge conservation causes the relation $I_{\alpha={\rm A/B}} = I_{\alpha,{\rm inj}} - I_{\rm T}$ between different currents. The cross correlation is decomposed,

$$\langle \delta I_{\rm A} \delta I_{\rm B} \rangle = - \left\langle \delta I_{\rm T}^2 \right\rangle + \langle \delta I_{\rm A,inj} \delta I_{\rm T} \rangle - \langle \delta I_{\rm B,inj} \delta I_{\rm T} \rangle + \langle \delta I_{\rm A,inj} \delta I_{\rm B,inj} \rangle . \tag{S19}$$

$\langle \delta I_{\rm A,inj} \delta I_{\rm B,inj} \rangle$ is nonzero when anyons are fractionalized into upstream and downstream parts upon tunneling at $\mathrm{QPC_C}$, but it is negligibly small when $\mathrm{QPC_C}$ is in the weak tunneling regime (see Sec. Supplementary Note 4).

We prove that for the case of $\mathrm{Im}[M] \neq 0$, the zero-frequency correlation $\langle \delta I_{\alpha,{\rm inj}} \delta I_{\rm T} \rangle$ between the injection current at $\mathrm{QPC}_{\alpha={\rm A,B}}$ and the tunneling current at $\mathrm{QPC_C}$ satisfies

$$\langle \delta I_{\alpha,{\rm inj}} \delta I_{\rm T} \rangle = e^* I_{\alpha,{\rm inj}} \frac{\partial I_{\rm T}}{\partial I_{\alpha,{\rm inj}}}. \tag{S20}$$

The case of $\mathrm{Im}[M] = 0$ will be discussed separately. We apply the perturbative expansion, over arbitrary orders of the tunneling strength at $\mathrm{QPC_A}$ and the lowest order of the tunneling strength at $\mathrm{QPC_C}$, to the correlation,

$$\langle \delta I_{\rm A,inj} \delta I_{\rm T} \rangle = \frac{1}{2} \int dt_1 \, \langle \{ \delta I_{\rm A,inj}(t_1), \delta I_{\rm T}(0) \} \rangle = \frac{1}{4} \int dt_1 \sum_{\eta_0,\eta_1=\pm} \langle T_K \{ \delta I_{\rm T}(0^{\eta_0}) \delta I_{\rm A,inj}(t^{\eta_1}) \} \rangle \equiv \sum_n \langle \delta I_{\rm A,inj} \delta I_{\rm T} \rangle_n \, . \tag{S21}$$

In the second equality, we expressed the anti-commutator using the Keldysh ordering. $\langle \delta I_{\rm A,inj} \delta I_{\rm T} \rangle_n$ represents the term of the order of $|\gamma_{\rm A}|^{2n} |\gamma_{\rm C}|^2$. Employing the Keldysh method, we obtain

$$\begin{aligned}
\langle \delta I_{\rm A,inj} \delta I_{\rm T} \rangle_n &= \frac{c_n}{4} \sum_{\eta_0,\eta,\eta_j} \eta \int dt dt_1 \cdots dt_{2n} \langle T_K \{ I_{\rm T}(0^{\eta_0}) H_{\rm T}(t^\eta) I_{\rm A,inj}(t_1^{\eta_1}) \prod_{j=2}^{2n} \eta_j H_{\rm A,inj}(t_j^{\eta_j}) \} \rangle - \int dt' \, \langle I_{\rm A,inj}(t') \rangle \, \langle I_{\rm T}(0) \rangle_{n-1} \\
=& \frac{c_n}{4} \frac{(2n-1)!}{n!(n-1)!} (-ie^*) \sum_{\eta_0,\eta,\eta_j=\pm} \eta \prod_{j=2}^{2n} \eta_j \int dt dt_1 \cdots dt_{2n} \Big[\langle T_K \{ I_{\rm T}(0^{\eta_0}) H_{\rm T}(t^\eta) \mathcal{A}(t_1^{\eta_1}) \mathcal{A}^\dagger(t_2^{\eta_2}) \prod_{i=2}^{n} \mathcal{A}^\dagger(t_{2i-1}^{\eta_{2i-1}}) \mathcal{A}(t_{2i}^{\eta_{2i}}) \} \rangle \\
& - \langle T_K \{ I_{\rm T}(0^{\eta_0}) H_{\rm T}(t^\eta) \mathcal{A}^\dagger(t_1^{\eta_1}) \mathcal{A}(t_2^{\eta_2}) \prod_{i=2}^{n} \mathcal{A}^\dagger(t_{2i-1}^{\eta_{2i-1}}) \mathcal{A}(t_{2i}^{\eta_{2i}}) \} \rangle \Big] - \int dt' \, \langle I_{\rm A,inj}(t') \rangle \, \langle I_{\rm T}(0) \rangle_{n-1} \\
=& \frac{1}{4} \frac{e^*}{(n-1)!} \sum_{\eta_0,\eta=\pm} \eta \int dt \Bigg[(\mathrm{Re}[M-1] \frac{I_{\rm A,inj}}{e^*} |t| + i \, \mathrm{Im}[M-1] \frac{I_{\rm A,inj}}{e^*} t)^{n-1} \Big[\sum_{\eta_1,\eta_2=\pm} \eta_2 \int dt_1 dt_2 \\
& \times \big[- \left\langle T_K \{ \mathcal{T}^\dagger(0^{\eta_0}) \mathcal{T}(t^\eta) \mathcal{A}(t_1^{\eta_1}) \mathcal{A}^\dagger(t_2^{\eta_2}) \} \right\rangle + \left\langle T_K \{ \mathcal{T}^\dagger(0^{\eta_0}) \mathcal{T}(t^\eta) \mathcal{A}^\dagger(t_1^{\eta_1}) \mathcal{A}(t_2^{\eta_2}) \} \right\rangle \big] - 2 \frac{I_{\rm A,inj}}{e^*} \int dt' \left\langle T_K \{ \mathcal{T}^\dagger(0^{\eta_0}) \mathcal{T}(t^\eta) \} \right\rangle \Big] \\
& - (\mathrm{Re}[M-1] \frac{I_{\rm A,inj}}{e^*} |t| - i \, \mathrm{Im}[M-1] \frac{I_{\rm A,inj}}{e^*} t)^{n-1} \Big[\sum_{\eta_1,\eta_2=\pm} \eta_2 \int dt_1 dt_2 \\
& \times \big[- \left\langle T_K \{ \mathcal{T}(0^{\eta_0}) \mathcal{T}^\dagger(t^\eta) \mathcal{A}(t_1^{\eta_1}) \mathcal{A}^\dagger(t_2^{\eta_2}) \} \right\rangle + \left\langle T_K \{ \mathcal{T}(0^{\eta_0}) \mathcal{T}^\dagger(t^\eta) \mathcal{A}^\dagger(t_1^{\eta_1}) \mathcal{A}(t_2^{\eta_2}) \} \right\rangle \big] - 2 \frac{I_{\rm A,inj}}{e^*} \int dt' \left\langle T_K \{ \mathcal{T}(0^{\eta_0}) \mathcal{T}^\dagger(t^\eta) \} \right\rangle \Big] \Bigg] \\
=& \frac{e^*}{(n-1)!} \int_{-\infty}^{\infty} dt \left\langle \left[\mathcal{T}^\dagger(0), \mathcal{T}(t) \right] \right\rangle_{\rm eq} (\mathrm{Re}[M-1] \frac{I_{\rm A,inj}}{e^*} |t| + i \, \mathrm{Im}[M-1] \frac{I_{\rm A,inj}}{e^*} t)^n .
\end{aligned} \tag{S22}$$

Here $c_n = (-i)^{2n}/(2n-1)!$, $I_{\rm A,inj} = -ie^*(\mathcal{A} - \mathcal{A}^\dagger)$, $H_{\rm A,inj} = \mathcal{A}(t) + \mathcal{A}^\dagger(t)$, $I_{\rm T} = -ie^*(\mathcal{T} - \mathcal{T}^\dagger)$, $H_{\rm T} = \mathcal{T} + \mathcal{T}^\dagger$. In the third equality, the approximation valid at large $V_{\rm inj}$ is applied as in Sec. Supplementary Note 1 so that the $n!$ equivalent ways of pairing $t_{2i-1} \simeq t_{2i}$ are considered. The following was used in computing the last disconnected term of $\int dt' \, \langle I_{\rm A,inj}(t') \rangle \, \langle I_{\rm T}(0) \rangle_{n-1}$,

$$\begin{aligned}
\langle I_{\rm T}(0) \rangle_{n-1} = \frac{1}{2(n-1)!} \sum_{\eta,\eta_0} \eta \int dt \Bigg[& (\mathrm{Re}[M-1] \frac{I_{\rm A,inj}}{e^*} |t| + i \, \mathrm{Im}[M-1] \frac{I_{\rm A,inj}}{e^*} t)^{n-1} \left\langle T_K \{ \mathcal{T}^\dagger(0^{\eta_0}) \mathcal{T}(t^\eta) \} \right\rangle \\
& - (\mathrm{Re}[M-1] \frac{I_{\rm A,inj}}{e^*} |t| - i \, \mathrm{Im}[M-1] \frac{I_{\rm A,inj}}{e^*} t)^{n-1} \left\langle T_K \{ \mathcal{T}(0^{\eta_0}) \mathcal{T}^\dagger(t^\eta) \} \right\rangle \Bigg] .
\end{aligned} \tag{S23}$$

The diverging integral $\int dt'$ in the last term cancels (i.e., regularizes) divergence in other terms. After calculations, the fourth equality is expressed with the equlibrium correlator $\langle [\mathcal{T}^\dagger(0), \mathcal{T}(t)] \rangle_{\rm eq}$. Finally, we find $\langle \delta I_{\rm A,inj} \delta I_{\rm T} \rangle_n = e^* I_{\rm A,inj} \partial \langle I_{\rm T} \rangle_n / \partial I_{\rm A,inj}$. Collecting all the terms of different n, we prove Eq. (S20). We note that a similar result was derived for Laughlin Abelian anyons using a different method of a non-equilibrium bosonization in Ref. [S7].

In the case of non-Abelian anyons having $\mathrm{Im}[M] = 0$, we directly compute $\langle \delta I_{\rm A,inj} \delta I_{\rm T} \rangle_n$ and $\langle \delta I_{\rm A,inj} \delta I_{\rm T} \rangle$, following the way in Sec. Supplementary Note 2 B, and find that the leading non-vanishing order of $\langle \delta I_{\rm A,inj} \delta I_{\rm T} \rangle$ is $O\Big(I_{\rm A/B,inj}^{2\delta + h_a - 1} V_{\rm A/B,inj}^{-h_a}\Big)$. Hence, in this case $\langle \delta I_{\rm A,inj} \delta I_{\rm T} \rangle$ is negligible in comparison with $\langle \delta I_{\rm T}^2 \rangle$, as their ratio is $O\Big((\frac{\hbar}{e^{*2}} \frac{I_{\rm A/B,inj}}{V_{\rm A/B,inj}})^{h_a} \Big)$ as in Eq. (S18).

Supplementary Note 4. SIDE EFFECTS BY ANYONS OF THE OPPOSITE CHIRALITY

At certain filling factors, there appear downstream charge modes and upstream neutral modes together on quantum Hall edges. In quantum Hall systems such as the anti-Pfaffian, particle-hole Pfaffian, and anti-Read-Rezayi states, it is expected that low-energy excitations of the upstream neutral modes are described by non-Abelian anyons. Here we show that our result is directly applicable to the situation where downstream modes and upstream modes coexist on edges, when the collider $\mathrm{QPC_C}$ is in the weak anyon tunneling regime.

Figure 2 shows a collider for studying upstream neutral-mode anyons. This setup corresponds to that of Fig. 1(a) in the main text, but having different locations of the sources and drains so that upstream neutral modes propagate from $\mathrm{QPC_{A/B}}$ to $\mathrm{QPC_C}$ on Edge A/B; in Fig. 1(a) downstream charge modes flow from $\mathrm{QPC_{A/B}}$ to $\mathrm{QPC_C}$ in a reversed magnetic field. It is possible to obtain information of the neutral modes from measurements of the charge currents and noises, since tunneling of neutral modes at QPCs is always accompanied by tunneling of charge modes.

When upstream and downstream modes coexist, a "back-action" happens. In Fig. 2, neutral-mode anyons are injected to Edge A/B at $\mathrm{QPC_{A/B}}$ and propagate to $\mathrm{QPC_C}$. When tunneling of a neutral-mode anyon happens at $\mathrm{QPC_C}$, it is accompanied by tunneling of a charge-mode anyon. This charge-mode anyon propagates backward from $\mathrm{QPC_C}$ to $\mathrm{QPC_{A/B}}$, and it affects the injection current $I_{\rm A/B,\,inj}$ at $\mathrm{QPC_{A/B}}$. In our parameter regime of $e^* V_{\alpha,\rm inj}/\hbar \gg I_{\alpha,\rm inj}/e^* \gg I_{\rm T}/e^*$, this back-action effect is negligible in comparison with $I_{\rm T}$ and $\langle \delta I_{\rm T}^2 \rangle$. It is because the back-action results in an effective additional bias voltage of order of $2\pi\hbar I_{\rm T}/e^{*2}$ across $\mathrm{QPC_{A/B}}$ which is much smaller than $V_{\alpha,\rm inj}$.

For concreteness, we compute the injection current $I_{\alpha,\rm inj}$ at $\mathrm{QPC}_{\alpha=\rm A,B}$ in the presence of $\mathrm{QPC_C}$. The back-action appears in the perturbation order $|\gamma_{\rm C}|^2$ of tunneling at $\mathrm{QPC_C}$ (while all the tunneling orders at QPC_α are considered),

$$I_{\alpha,\rm inj} = \sum_{n=0}^{\infty} \frac{(-i)^{2n+3}}{4(2n+1)!} \sum_{\eta_0,\eta,\eta_a,\eta_b=\pm} \eta\eta_a\eta_b \int dt dt_a dt_b \prod_{\eta_j} \eta_j \int dt_j \langle T_K \{ I_{\alpha,\rm inj}(0^{\eta_0}) H_{\alpha,\rm inj}(t^\eta) H_{\rm T}(t_a^{\eta_a}) H_{\rm T}(t_b^{\eta_b}) \prod_{j=1}^{2n} H_{\alpha,\rm inj}(t_j^{\eta_j}) \} \rangle. \tag{S24}$$

The integral over t_j's is done at large $V_{\alpha,\rm inj}$ as in Sec. Supplementary Note 1. Expanded the integrand around $t \simeq 0$, we find

$$I_{\alpha,\rm inj} \simeq \frac{e^* |\gamma_\alpha^2|}{d_\psi} \sum_{\eta=\pm} \eta \int dt \frac{e^{-ie^* V_{\rm inj} t/\hbar}}{[\epsilon - i\eta t]^{2\delta}} (1 \mp i \frac{2\pi\delta_{\rm ch}}{e^*} I_{\rm T} t). \tag{S25}$$

$\delta_{\rm ch}$ is the tunneling exponent of the downstream charge mode part. The second term is the back-action effect. Its sign factor $-$ (resp. $+$) is for $\alpha = \rm A$ (resp. B). We finally obtain

$$I_{\alpha,\rm inj} \simeq \frac{2\pi e^* |\gamma_\alpha|^2}{d_\psi} \Big[\frac{1}{\Gamma(2\delta)} (\frac{e^* V_{\rm inj}}{\hbar})^{2\delta-1} \pm \frac{1}{\Gamma(2\delta-1)} (\frac{e^* V_{\rm inj}}{\hbar})^{2\delta-2} \frac{2\pi\delta_{\rm ch}}{e^*} I_{\rm T} \Big]. \tag{S26}$$

The correction term is of the order of $I_{\alpha,\rm inj} \times \frac{I_{\rm T}/e^*}{e^* V_{\rm inj}/\hbar}$. It is hence negligible in the regime of $(e^*)^2 V_{\alpha,\rm inj}/\hbar \gg I_{\alpha,\rm inj} \gg I_{\rm T}$.

Supplementary References

[S1] Bonderson, P. Ph. D. thesis, California Institute of Technology, 2007.

[S2] Fendley, P., Fisher, M. P. A., & Nayak, C. Edge states and tunneling of non-Abelian quasiparticles in the $\nu = 5/2$ quantum Hall state and $p + ip$ superconductors, Phys. Rev. B **75**, 045317 (2007).

[S3] Francesco, P. Di, Mathieu, P., & Senechal, D. Conformal Field Theory (Springer, New York, 1997).

[S4] The (1+1)D holomorphic CFT correlator in any order can be obtained from (2+0)D holomorphic CFT with infinitesimal ϵ prescription. For the correlators $\langle \cdots O_i(x_i, t_i) \cdots O_j(x_j, t_j) \cdots \rangle = \lim_{\epsilon_i \to 0} \langle \cdots O_i(z_i) \cdots O_j(z_j) \cdots \rangle$ with $z_i = \epsilon_i + i(vt_i - x_i)$, and $\epsilon_i > \epsilon_j$ if O_i proceeds O_j in the correlator; see, e.g., Hartman, T., Jain, S., & Kundu, S. Causality Constraints in Conformal Field Theory, J. High Energy Phys. 05, 099 (2016). Hence, the ordering of the operators give rise to the additional dimension. If we place the coordinates of the operators in the complex plane and perform the braiding across the real axis, its direction is determined by whether $vt_i - x_i$ is larger than $vt_j - x_j$.

[S5] Han, C., Park, J., Gefen, Y., & Sim, H.-S. Topological vacuum bubbles by anyon braiding. Nat. Commun. **7**, 11131 (2016).

[S6] Lee, B., Han, C., & Sim, H.-S. Negative Excess Shot Noise by Anyon Braiding, Phys. Rev. Lett. **123**, 016803 (2019).

[S7] Rosenow, B., Levkivskyi, I. P., & Halperin, B. I. Current Correlations from a Mesoscopic Anyon Collider, Phys. Rev. Lett. **116**, 156802 (2016).
